# Extract of Kuding Tea Prevents High-Fat Diet-Induced Metabolic Disorders in C57BL/6 Mice via Liver X Receptor (LXR) β Antagonism

**DOI:** 10.1371/journal.pone.0051007

**Published:** 2012-12-04

**Authors:** Shengjie Fan, Yu Zhang, Na Hu, Qinhu Sun, Xiaobo Ding, Guowen Li, Bin Zheng, Ming Gu, Feisi Huang, Yin-Qiang Sun, Zhiqin Zhou, Xiong Lu, Cheng Huang, Guang Ji

**Affiliations:** 1 Drug Discovery Lab, School of Pharmacy, Shanghai University of Traditional Chinese Medicine, Shanghai, China; 2 Scientific Experimental Center, Shanghai University of Traditional Chinese Medicine, Shanghai, China; 3 Institute of Chinese Materia Medica, Shanghai University of Traditional Chinese Medicine, Shanghai, China; 4 College of Horticulture and Landscape Architecture, Southwest University, Chongqing, China; 5 Key Laboratory of Horticulture Science for Southern Mountainous Regions, Ministry of Education, Chongqing, China; 6 Institute of Digestive Disease, Longhua Hospital, Shanghai University of Traditional Chinese Medicine, Shanghai, China; University of Padova, Italy

## Abstract

**Objective:**

To investigate the effects of ilex kudingcha C. J. Tseng (kuding tea), a traditional beverage in China, on the metabolic disorders in C57BL/6 mice induced by high-fat diets.

**Design:**

For the preventive experiment, the female C57BL/6 mice were fed with a standard diet (Chow), high-fat diet (HF), and high-fat diet mixed with 0.05% ethanol extract of kuding tea (EK) for 5 weeks. For the therapeutic experiment, the C57BL/6 mice were fed high-fat diet for 3 months, and then mice were split and EK was given with oral gavages for 2 weeks at 50 mg/day/kg. Body weight and daily food intake amounts were measured. At the end of treatment, the adipocyte images were assayed with a scanning electron microscope, and the fasting blood glucose, glucose tolerance test, serum lipid profile and lipids in the livers were analyzed. A reporter gene assay system was used to test the whether EK could act on nuclear receptor transcription factors, and the gene expression analysis was performed with a quantitative PCR assay.

**Results:**

In the preventive treatment, EK blocked the body weight gain, reduced the size of the adipocytes, lowered serum triglyceride, cholesterol, LDL-cholesterol, fasting blood glucose levels and glucose tolerance in high-fat diet-fed C57BL/6 mice. In the therapeutic treatment, EK reduced the size of the white adipocytes, serum TG and fasting blood glucose levels in obese mice. With the reporter assay, EK inhibited LXRβ transactivity and mRNA expression of LXRβ target genes.

**Conclusion:**

We observed that EK has both preventive and therapeutic roles in metabolic disorders in mice induced with high-fat diets. The effects appear to be mediated through the antagonism of LXRβ transactivity. Our data indicate that kuding tea is a useful dietary therapy and a potential source for the development of novel anti-obesity and lipid lowering drugs.

## Introduction

Obesity is a worldwide problem and its prevalence is increasing rapidly [1]. Obesity is caused by the storage of excess calories as triglycerides in adipose tissue and abnormally in other tissues [2], which is associated with insulin resistance, type 2 diabetes, hypertension, hyperlipidemia, cardiovascular disease, stroke and non-alcoholic steatohepatitis [3], [4]. The prevention and treatment of obesity will greatly benefit patients with the disease. Currently there is only one approved drug, (Orlistat) by the FDA for long-term use in the treatment of obesity. Therefore, new therapeutic approaches are urgently required for the treatment of obesity [3].

Liver X receptors (LXRs) are members of the nuclear receptor family of transcription factors. Two isoforms of LXR, LXRα and LXRβ, have been identified, and they are important regulators of lipids and cholesterol homeostasis. LXRα knockout mice are healthy when fed a low-fat diet. However, LXRα knockout mice develop high cholesterol levels in the liver and enlarge fatty livers when fed a high-fat diet [5]. LXRβ knockout mice are unaffected by a high-fat diet, suggesting that LXRα and LXRβ have different roles [6].

LXRs are potential drug targets for obesity, dyslipidemia and atherosclerosis. Previous work has shown that the synthetic LXR agonist GW3965 lowers cholesterol levels in both serum and the liver, inhibits the development of atherosclerosis in mouse models [7], [8], and improves glucose tolerance in diet-induced obesity and insulin resistant mice by regulating genes involved in glucose metabolism in the liver and the adipose tissue [9]. However, GW3965 increases triglycerides levels of plasma and livers in mice. On the other hand, LXR antagonists, such as 5α, 6α-epoxycholesterol-3-sulfate, block the formation of plaques of atherosclerosis by inhibiting LXR function [10]. Developing new potent and effective LXR agonists and antagonists without the side-effects may be beneficial for clinical use.

Green tea and kuding tea are two of the most popular beverages in China. Green tea has been well studied for its various health benefits, but there is little data on the biological activities of bitter tea. Kuding tea has been used in China for more than 2000 years as a beverage. In traditional Chinese medicine, kuding tea has also been used in the formulae for treating obesity, hypertension, cardiovascular disease, hyperlipidemia and various other diseases. Recently, several clinical studies have focused on its effects on lipid lowering, body weight reduction and blood glucose lowering in patients with metabolic syndromes. Animal studies have shown that the phenolic constituents and phenylethanoid glycosides of kuding tea exhibit significant antioxidant activities *in vitro* [11], [12]. In addition, kuding tea also significantly reduces middle cerebral artery occlusion and reperfusion (MCAO/reperfusion), induces infarction and neurological deficits and loss of neural cells, and inhibits the phosphorylation of mitogen-activated protein kinase and cyclooxygenase-2. It also increases anti-apoptotic protein levels in MCAO/reperfusion rat brains [13]. The total saponins from ilex kuding tea improve abnormal hemorheological parameters in ApoE^−/−^ mice induced by a high-fat diet [14]. Triterpenoid saponins from the leaves of ilex kuding tea inhibit the LDL-induced formation of foam cells and reduce intracellular total cholesterol and triglyceride contents [15]. However, there is no solid experimental data to support the effects of kuding tea on obesity and hyperlipidemia. Here, we show that extracts of kuding tea prevent the development of obesity, hyperlipidemia and glucose tolerance in high-fat diet-fed C57BL/6 mice, and inhibits the transactivities of LXRβ.

## Materials and Methods

### Fingerprint Analysis by Reverse Phase High Performance Liquid Chromatography (RP-HPLC)

To analyze the fingerprint profiles, EK (5 mg) was dissolved in methanol (1 ml). Filtered extracts were analyzed using an Agilent 1200 liquid chromatograph system with a UV detector at the λ _max_ of 270 nm. Chromatographic separation was performed on Discovery C-18 reverse phase column (250×4.6 mm, 5 µm) with an injection volume of 10 µl methanol (as solvent A) and water (as solvent B). The gradient was set as follows: 0∼10 min, 5% B; 20 min, 30% B; 25 min, 50% B; 40 min, 90% B; 45 min, 95% B (flow rate 1 ml/min).

Ursolic acid and Lupeol (purity >98%) were purchased from Shanghai R&D Center for standardization of Chinese Medicines (Shanghai, China). The compounds were monitored at 210 nm using a Discovery C18 Column with methanol or acetonitrile (as solvent A) and water containing 0.1% phosphoric acid (as solvent B) in the mobile phase at a flow rate of 1.0 mL/min at 30°C for 60 min. To detect Ursolic acid, the gradient elution of HPLC was 48% A (acetonitrile) and 52% B (phosphoric acid, pH 2.5) at 0 min, 75% A and 25% B at 40 min, 85% A and 15% B at 60 min. The constant mobile phase of methanol: water (98∶2, v/v) was used for detection of lupeol.

### Cell Culture

3T3-L1 cells were grown and maintained in DMEM containing 10% fetal bovine serum (Hyclone, Logan, UT). For adipocyte differentiation, cells were grown in 12-well plates to full confluence for 2 days and then differentiation medium (DM) containing 10 µg/ml insulin (Sigma, St. Louis, MO), 0.5 µM dexamethasone (Sigma, St. Louis, MO), and 0.8 mM isobutylmethyl xanthine (IBMX, Sigma, St. Louis, MO) was added to the culture. After 4 days, the medium was changed to DMEM with 10% fetal bovine serum for differentiation at 37°C in 10% CO_2_. EK was dissolved in DMSO and was added to the medium at indicated concentrations. DMSO was added to the cells as the untreated control.

### Oil Red O Staining

The cells were washed with PBS twice, fixed with 10% formalin at room temperature for 10 minutes, and then stained with oil red O (Sigma, St. Louis, MO) at 60°C for 10 minutes. Pictures were then taken using an Olympus (Tokyo, Japan) microscope.

### Quantitative Real-time PCR

Total RNA was extracted using a spin column (Qiagen, Hilden, Germany) according to the manufacturer’s instructions, and RNA was treated with DNase I to remove genomic DNA contamination. The first strand cDNA was synthesized using a cDNA synthesis kit (Fermentas, Madison, WI), and gene expression levels were analyzed by quantitative real-time RT-PCR using the ABI Stepone Plus Real Time PCR system (Applied Biosystems, Carlsbad, CA). The primers used in the experiments are shown in Table 1. The mRNA levels of all genes were normalized using β-actin as internal control.

**Table 1:** Sequences of the primers used in real-time PCR.

Gene	Forward primer	Reverse primer
β-Actin	TGTCCACCTTCCAGCAGATGT	AGCTCAGTAACAGTCCGCCTAGA
LXRα	GAGTGTCGACTTCGCAAATGC	CCTCTTCTTGCCGCTTCAGT
LXRβ	CAGGCTTGCAGGTGGAATTC	ATGGCGATAAGCAAGGCATACT
ABCA1	GGCAATGAGTGTGCCAGAGTTA	TAGTCACATGTGGCACCGTTTT
ABCG1	TCCCCACCTGTAAGTAATTGCA	TCGGACCCTTATCATTCTCTACAGA
ApoE	GAACCGCTTCTGGGATTACCT	TCAGTGCCGTCAGTTCTTGTG
Cyp7a1	GTGGTAGTGAGCTGTTGCATATGG	CACAGCCCAGGTATGGAATCA
SREBP1	GGCTATTCCGTGAACATCTCCTA	ATCCAAGGGCATCTGAGAACTC
FAS	CTGAGATCCCAGCACTTCTTGA	GCCTCCGAAGCCAAATGAG
LPL	ATCGGAGAACTGCTCATGATGA	CGGATCCTCTCGATGACGAA
C/EBPβ	GGGGTTGTTGATGTTTTTGG	CGAAACGGAAAAGGTTCTCA
PPARα	AGGCTGTAAGGGCTTCTTTCG	GGCATTTGTTCCGGTTCTTC
PPARγ	CGCTGATGCACTGCCTATGA	AGAGGTCCACAGAGCTGATTCC
PPARβ/δ	AGTGACCTGGCGCTCTTCAT	CGCAGAATGGTGTCCTGGAT
C/EBPα	CGCAAGAGCCGAGATAAAGC	CACGGCTCAGCTGTTCCA
aP2	CATGGCCAAGCCCAACAT	CGCCCAGTTTGAAGGAAATC
ACC	GAATCTCCTGGTGACAATGCTTATT	GGTCTTGCTGAGTTGGGTTAGCT
ACO	CAGCACTGGTCTCCGTCATG	CTCCGGACTACCATCCAAGATG
UCP-2	GGGCACTGCAAGCATGTGTA	TCAGATTCCTGGGCAAGTCACT

### Transfection of Cultured Cells and Reporter Assays

The reporter assay was carried out as previously described [16], [17]. Briefly, 293T cells were grown in 24 well plate for transfection. The expression plasmid pCMXGal-mouse peroxisome proliferator-activated receptor (PPAR) α, γ, β/δ, LXRα and LXRβ-LBD, and the Gal4 reporter vector MH100 × 4-TK-Luc were gifts from Dr. R Evans [18] and Dr. Saez [19], [20]. When expression plasmids were co-transfected with a reporter construct, 1 µg of the relevant plasmid was combined with 1 µg of reporter plasmids and 0.1 µg of the pREP7 (rellina luciferase) reporter to normalize transfection efficiencies. All transfections included 2.1 µg of total plasmids and 5 µl FuGENE HD (Roche, Germany) per ml of DMEM. Transfection solution was added to 293T cells for 24 hours and then removed, and 10 µM of PPARγ, PPARα, PPARβ/δ and LXR agonist rosiglitazone, WY14643, GW0742 and GW3965 or EK were added, before the cells were harvested for determination of luciferase activity 24 hours later. The luciferase reporter assays were carried out using the Dual-Luciferase Reporter Assay System (Promega, San Luis Obispo, CA), and the transfection efficiencies were normalized according to rellina luciferase activity. All of the transfection experiments were performed in triplicate independently.

### Animals and Serum Chemistry Analysis

The animal protocols used in this study were approved by the Shanghai University of Traditional Chinese Medicine for animal studies (Approved Nember:11002). Female C57BL/6 mice were purchased from the SLAC Laboratory (Shanghai, China). All mice were kept under controlled temperatures (22–23°C) and on a 12-hour light, 12-hour dark cycle. For the preventive experiment, C57BL/6 mice of similar ages and body weights were randomly divided into different groups, and then placed on a high fat (HF) diet (60% of calories derived from fat, Research Diets, New Brunswick, NJ; D12492), or on a high-fat diet mixed with 0.05% of EK, or on a low-calorie diet as the equivalent chow diet control (10% of calories derived from fat, Research Diets; D12450B). The diet study was started at 6 weeks of age and continued for 11 weeks. For the therapeutic experiment, the mice were placed on a high fat diet for 3 months and then the obese mice were grouped randomly. EK was given with oral gavages for 2 weeks at 50 mg/day/kg (HF +EK) while the control mice were given water with oral gavages (HF). The normal control mice were kept on the normal diet through the experiment (Chow). Twenty-four hour food intake was measured by recording the difference in weight between the food put into the cage and that remaining at the end of 24 hours in both treated groups and controls. The experimental diets did not result in any change in the daily food intake compared with controls. Serum triglyceride (TG), total cholesterol (TC), HDL cholesterol (HDL-c), and LDL cholesterol (LDL-c) levels were examined using a Hitachi 7020 Automatic Analyzer (Hitachi, Tokyo, Japan) with 100 µl of heart blood serum.

### Liver and Fecal Lipid Content Analysis

The liver samples were weighed and homogenized in tissue lysis buffer (20 mM Tris·HCl pH 7.5, 150 mM NaCl, 1% Triton) and extracted with an equal volume of chloroform. The chloroform layers were dried and dissolved in isopropyl alcohol to measure lipid levels as described above. Fecal lipids were also extracted and measured as described above.

### Hematoxylin and Eosin (H&E) Staining

For H&E staining, the tissue was fixed in 10% formaldehyde, embedded in OCT compound and cut into 10 µm section according to a standard protocol. The sections were stained with Hematoxylin and eosin and examined under a light microscope.

### Scanning Electron Microscopy

Scanning electron microscopy was used to examine the structure of fat tissue according to the previously described protocols [21]. The images were taken using a Philip XL-30 scanning electron microscope.

### Intraperitoneal Glucose Tolerance Test

After 2 weeks of treatment, C57BL/6 mice were fasted overnight for 12 hours. The blood samples were collected from the tail vein for determination of baseline glucose values (0 minutes) before the injection of glucose (1 g/kg body weight). Additional blood samples were collected at regular intervals (15, 30, 60, and 90 minutes) for glucose measurement.

### Statistical Analysis

Data analyses were performed using the SPSS12.0 for Windows statistical program. All data were presented as means ± SE. Statistical analysis was done by one-way analysis of variance (ANOVA). Differences were considered significant when P<0.05.

## Results

### Fingerprint Profiling of EK

To analyze the components of the ethanol extract of kuding tea, we assayed the fingerprint of EK by HPLC. Figure S1A showed that EK contains multiple peaks consistent with the previous findings. We further compared the elements of EK to known compounds ursolic acid and lupeol. Figure S1B showed that there was an absorption peak at 41.147 min of the retention time which was same as the peak of ursolic acid (Figure S1C). Similarly, a peak of EK at the retention time of 17.379 min (Figure S1D) was identity to lupeol (Figure S1E). These findings are consistent with the previous report [22].

### EK Inhibits 3T3-L1 Adipocyte Differentiation

**Figure 1:**
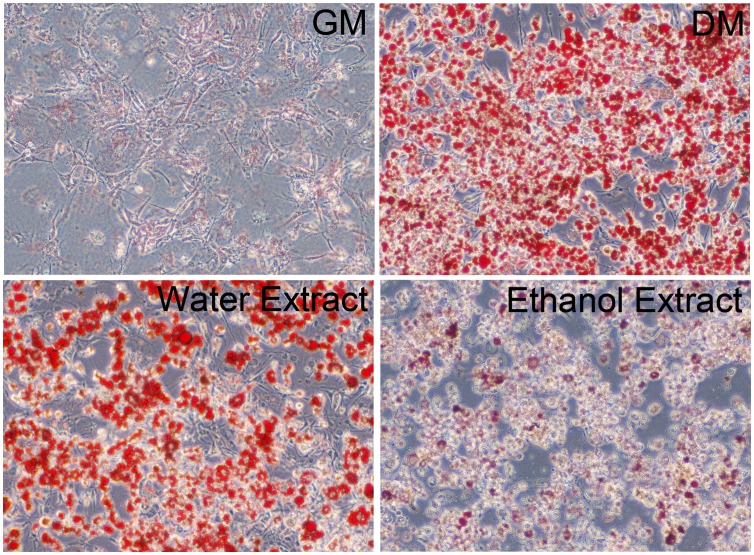
The ethanol extract of kuding tea inhibits 3T3-L1 adipocyte differentiation. The water extract and the ethanol extract were added into the medium at the concentration of 20 µg/ml. DMSO was used as the vehicle control. The cells were stained with oil red O at day 6 of differentiation. GM: growth medium; DM: differentiation medium.

Since kuding tea has been used for the prevention and treatment of obesity and hyperlipidemia, we observed the effects of EK on the differentiation of 3T3-L1 adipocytes. We used insulin, dexamethasone, and isobutylmethyl xanthin (differentiation medium, DM) to induce 3T3-L1 pre-adipocyte differentiation. During the DM induction, EK was added to the medium from day 0 until day 6 of differentiation. The results showed that the ethanol extract of kuding tea inhibited the differentiation of 3T3-L1 adipocyte significantly, whereas the water extract of kuding tea did not alter 3T3-L1 adipocyte differentiation (Figure 1), indicating that the potential agent for the treatment of obesity and dyslipidemia may be liposoluble components. As a result, we used the ethanol extract in the following experiments.

### EK Inhibits Later Stage of 3T3-L1 Adipocyte Differentiation

**Figure 2:**
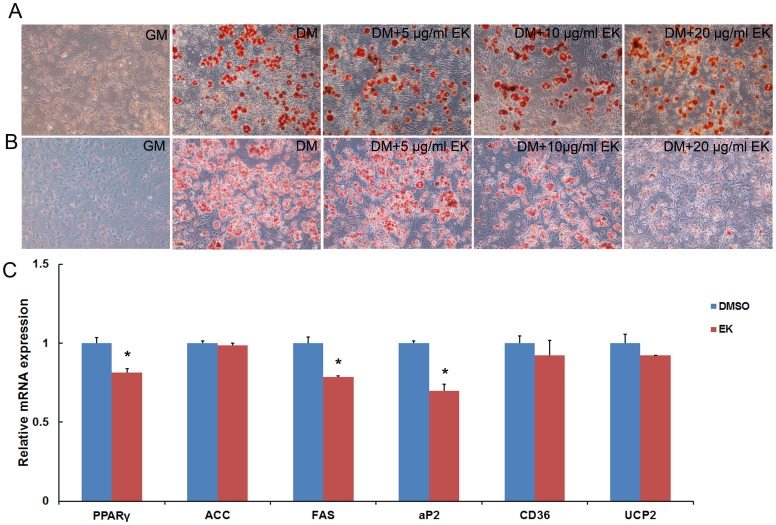
EK inhibits 3T3-L1 adipocyte differentiation induced by DM. (A): EK was used at the beginning of DM induction of 3T3-L1 cells and was removed during differentiation. (B): EK was only used during the differentiation of 3T3-L1 cells. The cells were stained with oil red O at day 6 of differentiation. (C): Real-time RT-PCR results of gene expression levels in 3T3-L1 adipocyte. The cell was differentiated for 6 days and then the cell was treated with EK at 20 µg/ml for 24 hours. DM: differentiation medium. β-actin was used as an internal control. Data are presented as means ± SE for 4 treatments. *P<0.05.

The differentiation of 3T3-L1 adipocytes involves two steps: a 4 day induction and 5–7 days of differentiation, both of which are controlled by different molecular events [23], [24], transcription factor activation and the lipogenic gene expression, respectively. To test which process of adipocyte differentiation EK inhibits, EK was added to the culture medium at different stages. When EK was added during induction and removed during differentiation, the differentiation was not obviouslychanged (Figure 2A). Interestingly, when EK was added after the completion of induction, the differentiation was inhibited significantly (Figure 2B), indicating that EK may disturb later stages of differentiation processes of 3T3-L1 adipocytes.

To confirm this, quantitative PCR was carried out to test the alterations of gene expression of related genes in the EK treated 3T3-L1 adipocyte. The results showed that EK significantly inhibited the expression of the adipocyte marker of PPARγ and aP2. The expression of fatty acid synthase (FAS) was also reduced following EK treatment. However, EK treatment did not alter the expression of ACC, CD36 and UCP2 genes (Figure 2C).

### EK Blocks the High-fat Diet-induced Obesity and Hyperlipidemia in C57BL/6 Mice

**Figure 3:**
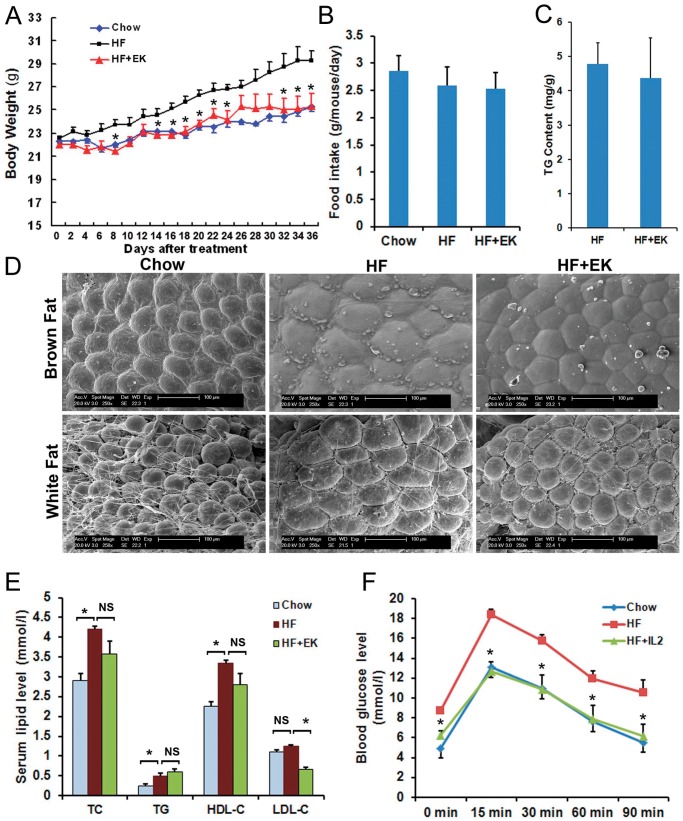
EK ameliorates metabolic disorders in high-fat diet-induced obesity C57BL/6 mice. (A): Body weight gain. (B): Food intake amount. (C): Fecal TG contents. (D): Images of white and brown adipocytes under a scanning electron microscope. (E): Serum total cholesterol (TC), triglyceride (TG), low-density lipoprotein cholesterol (LDL-c), and high-density lipoprotein cholesterol (HDL-c) levels. (F): Effects of EK on glucose tolerance in HFD fed mice as determined by the glucose tolerance test (GTT). NS: No significance. The data were shown as mean ± SE. N = 7 for all groups. * P<0.05.

We next examined the effects of EK on increased body weight induced by a high-fat diet in C57BL/6 mice. After 5 weeks of treatment, the body weight of the mice fed the high-fat diet was increased significantly when compared to that of the mice on the standard diet (chow). When supplemented with EK, the body weight gained was much less than in the HF control mice (Figure 3A). Since inhibition of body weight gain may be caused by less amount of food intake, we examined the food intake amounts of the mice. The EK did not suppress the food intake when compared to that in HF control mice (Figure 3B), indicating that the body weight reduction in EK treated mice was not as a result of the lower calorie intake. Next, we tested fecal TG content to assess whether EK inhibits the lipid absorption in the intestine. The results showed that the EK treatment did not alter the fecal TG contents (Figure 3C), which suggests that the role of EK in blocking body weight gain is not as a result of the inhibition of lipid absorption. Next, we examined the mass of adipocytes using a scanning electron microscope. The size of both white adipocytes and brown adipocytes were reduced significantly in comparison with the control adipocytes under the HF diet (Figure 3D), indicating that EK protects from the HF-induced increase in fat mass.

Obesity is closely associated with hyperlipidemia and insulin resistance, so we tested the serum lipid and fasting blood glucose levels in mice. Serum lipid analysis displayed that EK treatment led to a reduction of LDL-c levels under HF diet conditions, but TG, TC and HDL-c levels were not changed significantly (Figure 3E). Fasting glucose levels were lower than those in HF control mice (0 min in Figure 3F). We then examined whether EK treatment could affect glucose tolerance using a glucose tolerance test. The blood glucose levels were measured at 15, 30, 60, and 90 minute intervals following intra-peritoneal injections of 1 g/kg glucose. As shown in Figure 3F, the glucose levels in HF diet-fed mice were markedly higher than those in the chow diet fed mice. EK treatment significantly lowered the glucose levels at all of the time points, suggesting that EK improves the glucose tolerance of HF diet-fed C57BL/6 mice.

### EK Prevents Lipid Accumulation in the Liver of C57BL/6 Mice

**Figure 4:**
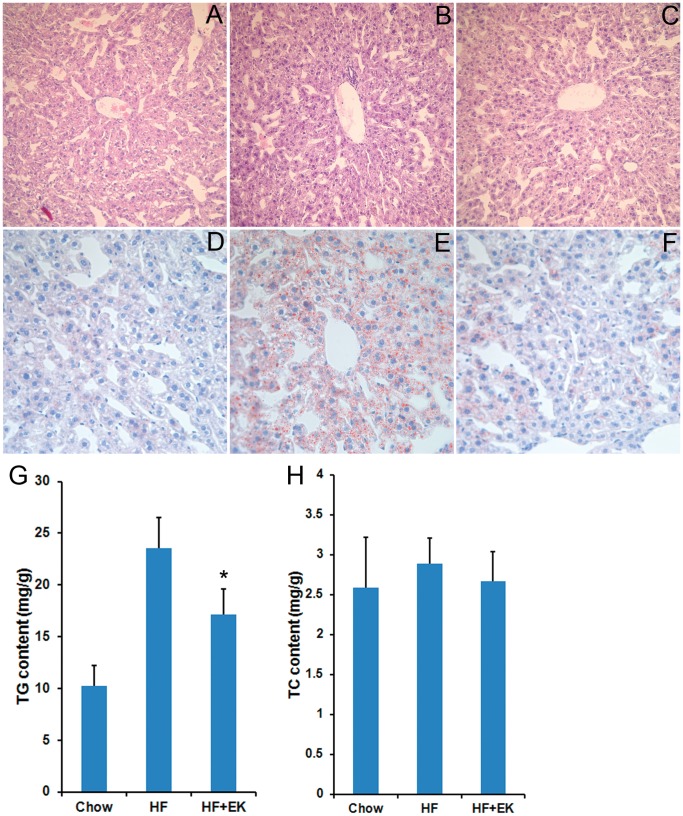
EK improves lipid accumulation in the liver of high-fat diet-induced C57BL/6 mice. (A-C): H&E staining (×200) of livers from the standard diet (A), HF diet (B) and HF+EK mice (C). (D-F): Oil red O staining (×400) of the liver sections from the standard diet (D), HF diet (E) and HF+EK mice (F). The sections were counterstained with hematoxylin. The quantitative results of TG (G) and TC (H) content in livers are shown. The mice were fed with a high-fat diet for 5 weeks and EK was powdered and mixed into the diet at 0.05% (wt/wt). The data were presented as mean ± SE. N = 7 for all groups. * P<0.05.

To assay the effects of EK on hepatic steatosis induced by a high-fat diet, we examined the fat content and lipid profile in the liver of EK-treated mice. HE staining showed that mice fed the high-fat diet for 5 weeks had similar morphologies of hepatocyte tissues to the standard diet-fed mice (Figure 4A-C). Oil red O staining showed lipids were accumulated in the liver of HF mice when compared to that in chow control mice (Figure 4D,E), and that EK treatment notably prevented the lipid accumulation in the liver (Figure 4F). To confirm these results, the liver TG and TC contents were analyzed. The TG levels in the HF diet-fed mice were markedly higher than that in the control mice, whereas EK treatment significantly prevented the TG accumulation in the liver induced by a high-fat diet (Figure 4G). Total TC contents in the liver did not show significant changes in all groups (Figure 4H). The results suggest that EK could prevent lipid accumulation and block the development of hepatic steatosis induced by high-fat diet in mice.

### EK Improves Metabolic Disorders in Obese Mice

**Figure 5:**
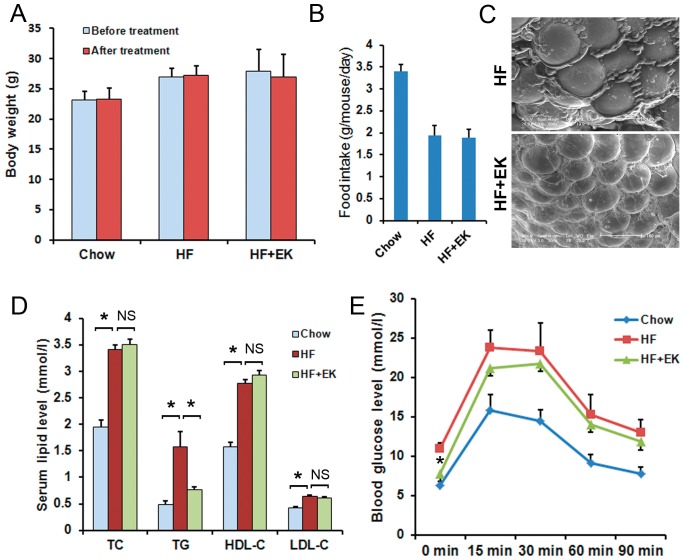
EK improves metabolic disorders in obese C57BL/6 mice. (A): Body weights before and after treatment. (B): Food intake amount. (C): Images of white adipocytes using scanning electron microscopy. (D): Serum total cholesterol (TC), triglyceride (TG), low-density lipoprotein cholesterol (LDL-c), and high-density lipoprotein cholesterol (HDL-c) levels. (E): Glucose tolerance in HFD fed mice as determined by glucose tolerance test (GTT). The mice were injected glucose at 1 mg/kg for the intra-peritoneal glucose tolerance test, and glucose levels were tested at regular intervals of 15, 30, 60, and 90 minutes. NS: No significance. The data were shown as mean ± SE. N = 7 for all groups. * P<0.05.

Next, we tested whether EK could affect the metabolic disorders in obese mice. After 3 months feeding of high-fat diet, C57BL/6 mice developed high body weight, serum lipids and blood glucose. Then the mice were divided into two groups and treated with 50 mg/kg/day EK for 2 weeks. EK treatment did not significantly reduce body weights (Figure 5A), and the food intake amount was not changed either (Figure 5B). However, EK treatment resulted in a reduction in the size of the white adipocytes in obese mice compared to those in control mice (Figure 5C).

Serum lipid analysis showed that TC, TG, LDL-c and HDL-c levels in obese mice were significantly increased when compared to those seen in standard diet fed mice. EK treatment notably lowered the TG contents, but did not change the serum TC, LDL-c or HDL-c levels (Figure 5D), showing different effects of EK on lipid profiles in the prevention and treatment of obesity mice.

Furthermore, we tested the fasting blood glucose levels and glucose tolerance in EK treated mice. Figure 5E shows that the fasting glucose levels in EK fed mice were lowered when compared to that of untreated mice, and glucose levels were also improved at 30 and 60 minutes following intra-peritoneal injections of glucose, suggesting that EK could ameliorate the glucose tolerance. Taken together, these results indicate that EK could alleviate the metabolic disorders in diet-induced obese mice.

### EK Inhibits the Lipogenic Gene Expression in the Mouse Liver

**Figure 6:**
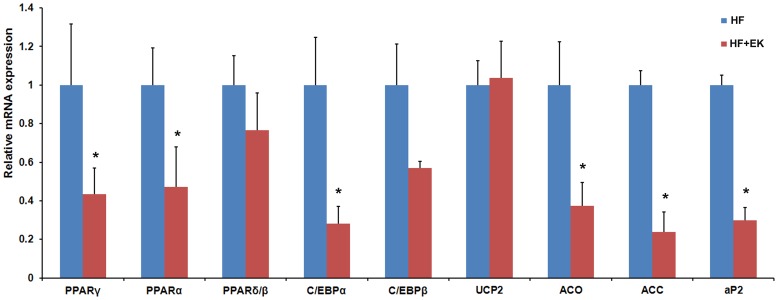
EK inhibits the expression of lipogenic genes in the mouse liver. 3T3-L1 adipocyte differentiation induced by DM. Real-time RT-PCR results of gene expression levels of PPARγ, PPARα, PPARδ/ß, C/EBPα, C/EBPß, UCP2, ACO, ACC and aP2 in liver of EK treated mice were compared to that of HF control mice from preventive treatment. β-actin was used as an internal control. Data are presented as means ± SE for 5 mice per group. *P<0.05.

To test the effects of EK in vivo, we examined the gene expression in the liver of EK treated mice. As shown in Figure 6, mRNA levels of transcription factors such as PPARγ, CEB/Pα, and lipogenic genes such as acyl-CoA oxidase (ACO), acetyl-coenzyme A carboxylase (ACC), and aP2 were decreased in EK treated livers, suggesting that the expression of lipogenic related genes is affected by EK treatment.

### Kuding Tea Contains the Ligand of LXRβ Antagonist

**Figure 7:**
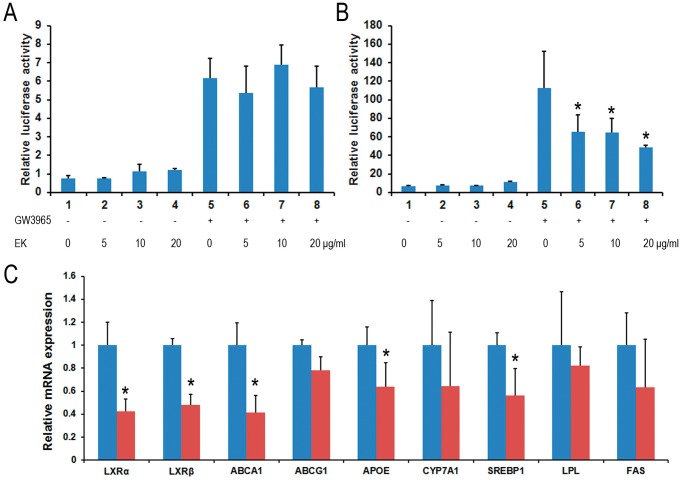
EK Nuclear receptor transcription activity assay. (A, B): LXRα and LXRβ trans-activities. The expression plasmids of pCMXGal-mouse LXRα and LXRβ-LBD were co-transfected with Gal4 reporter vector MH100 × 4-TK-Luc to 293T cell for 24 hours. Then the cell was treated with 10 µM of LXR agonist GW3965 and/or 5–20 µg/ml of EK for another 24 hours. DMSO was used as the vehicle control. The relative luciferase activities were measured by comparison to rellina luciferase activities. The results represent at least three independent experiments and data are presented as means ± SE. *P<0.05. (C) Gene expression levels of LXR target genes in livers of EK treated mice were compared to that of HF control mice from preventive treatment, and β-actin was used as an internal control. Data are presented as means ± SE for 5 mice per group. *P<0.05.

Nuclear receptor transcription factors are important regulators of lipid and glucose homeostasis. Based on the inhibition of obesity and hyperlipidemia, we tested whether EK acts on PPARγ, α, β/δ and LXRα and LXRβ, which are drug targets for metabolic syndromes [25], [26]. We did not observe the inhibitory effects of EK on the transactivities of PPARγ, α, β/δ (data not shown) or LXRα (Figure 7A). However, LXRβ transactivity induced by GW3965 was significantly inhibited by EK in a dose-dependent manner (Figure 7B), suggesting that EK may contain the ligand of LXRβ antagonist.

LXR regulates lipid and glucose metabolism through the activation of expression of a set of target genes, including fatty acid synthase (FAS), sterol regulatory element-binding protein-1c (SREBP-1c), lipoprotein lipase (LPL), ATP-binding cassette transporter A1 (ABCA1), ATP-binding cassette transporter G1 (ABCG1) and LXR itself [27]. We then evaluated the *in vivo* effect of EK on LXRβ activity related gene expression by analyzing the mRNA expression levels in the liver tissues isolated from EK treated and HF control mice. The levels of LXRα/β, ApoE, ABCA1 and SREBP1 were also inhibited in the livers of EK treated mice (Figure 7C), indicating that some components of kuding tea could act as LXRβ antagonist.

## Discussion

In this study, we provide the evidence that kuding tea could prevent and alleviate metabolic disorders in high-fat diet-fed mice. Our results clearly showed that EK treatment blocks the body weight gain, hyperlipidemia, and insulin resistance in the mice induced with HF diet. Chemical and histologic evidence showed that EK treatment resulted in a significant reduction of lipid accumulation in hepatic issues of DIO mice, suggesting that EK has protective effects against the development of metabolic disorders such as obesity, dyslipidemia, diabetes and hepatic steatosis in mice. We also found that EK could improve metabolic disorders in obese mice. Furthermore, we determined that EK selectively suppresses the transactivity of the nuclear receptor transcription factor LXRβ. Thus, the findings from this investigation suggest that the protective effect of EK against metabolic disorders is likely due to its inhibitory effect on LXRβ.

Kuding tea is a popular beverage in China. Like green tea, kuding tea is used in health care formulae to ameliorate metabolic disorders such as obesity. In recent years, kuding tea has been reported to have various biological effects [28]. It has been shown that kuding tea lowers serum TG, TC and LDL-c levels in dyslipidemia patients [29], [30], and improves blood pressure in phase I hypertension patients, as well as preventing the progression of atherosclerosis [31], [32].

C57BL/6 mice can develop metabolic syndromes when fed a high-fat diet. In the current study, the body weight, serum TC, TG, LDL-c and liver TG levels, glucose tolerance were significantly increased in mice fed a high-fat diet for 5 weeks. EK treatment resulted in a significantly lower weight gain and serum TC, and improved glucose tolerance and lipid accumulation in the liver, suggesting that EK could prevent the development of metabolic syndromes.

Weight gain is a consequence of the increase in adipocyte mass and numbers caused by excess calories stored as TG [33], while weight loss was usually caused by a reduction of the adipocyte mass and number by suppression of energy intake or overburn of excess calories. The specific mechanisms by which EK protects against weight gain is yet to be defined. There are several likely possibilities based on evidence available from our studies. Because a reduction in food intake may significantly affect body weight, blood glucose and lipid levels, we considered the possibility that the effect of EK may result from reduction of food intake. However, we did not observe the difference of food intake amount between the HF group and EK treatment mice. Green tea has been reported to inhibit intestinal lipid absorption [34]–[36]. However, our data showed that kuding tea does not have an inhibitory effect on the lipids absorption. Thus, it is probable that the decreased lipid levels and body weight may not be associated with the decreased absorption of lipids at an intestinal level. Taken together, our data indicate that the protective mechanisms of EK against body weight gain are independent of the reduction of energy intake and absorption of the lipids in the gut.

In our therapeutic experiment, the EK-treated obese mice displayed lower serum TG and fasting glucose levels than obese control mice. However, there is no significant body weight loss, TC or LDL-c reduction in the mice. There are two possible reasons for this discrepancy. First, removing of excess fat has been shown to be much more difficult than prevention of fat gain. Second, in the preventive therapy, we treated the mice for 5 weeks, but the mice were only treated for two weeks for therapeutic treatment. Therefore, the results of the present study do not support the weight-reducing effects of kuding tea in clinical trials reported by previous investigators. The glucose tolerancewas improved in preventive treatment, but was less effective in therapeutic treatments. This may result from the reduction of fat tissue because the deposition of TG in cells is responsive for the development of insulin resistance [37]–[39].

Compared to the water extract of kuding tea, the ethanol extract inhibited the adipocyte differentiation of 3T3-L1 adipocytes suggesting that liposoluble components of kuding tea may act on the adipocytes. The chemical analysis has shown that the ethanol extract of kuding tea contains 11 major compounds: lupeol, 11-keto-α-amyrin palmitate, α-amyrin palmitate, 12-ursene-3,28-diol, ursolic acid, 3β-hydroxylup-20(29)-en-30-al, 3β-hydroxy-20-oxo-30-norlupane, tanacetene, β-sitosterol, n-behenic acid and n-hexacosane [22]. Of these, ursolic acid has been studied for its effects on metabolic disorders. For example, ursolic acid enhances the binding of PPAR-α to PPRE, regulates the expression of lipid metabolism genes and significantly reduces intracellular triglyceride and cholesterol concentrations in hepatocytes [40], and decreases body weights, visceral adiposity, levels of blood glucose and plasma lipids, as well as increasing plasma leptin in high-fat diet-fed mice [41]-[43]. Lupeol has also been reported to lower blood glucose in experimental diabetic animals [44], [45]. However, in the extract used in current study, it only contains 6.48% of ursolic acid (about 1/20 used the previous studies), suggesting that ursolic acid is not the only effective component. The active compounds in kuding tea need to be investigated for their roles in metabolic diseases. Or an intestinal permeation system could be used to elucidate the mechanism of kuding tea on 3T3L1 adipocyte.

Published data regarding the mechanism of kuding tea are limited, but the studies suggest that kuding tea may improve metabolic disorders through multiple mechanisms. Our data suggest that kuding tea may prevent metabolic disorders by selectively targeting nuclear receptors of transcription factors LXRβ. The LXR family are ligand-activated transcription factors including both LXRα and LXRβ. LXRα is expressed primarily in the liver, adipose tissue, and macrophages, while LXRβ is ubiquitously expressed [5], [6]. The potential of the LXRs as drug targets for hyperlipidemia, AS, diabetes, hypertension and inflammation have been previously shown [46], [47]. Further investigation of the effects of EK with LXRβ knockout mice will confirm the signaling pathway of EK. And probably this will identify the inhibition of LXRβ as a therapy for metabolic diseases in vivo.

Previous studies have shown that LXR agonists could lower serum TC levels, but increase liver and serum TG levels, which excludes the LXR agonists as a therapy for metabolic diseases. Development of selective agonists or antagonists of LXRs may avoid the off-target effects [19]. Recently two naturally occurring compounds, rhein and naringenin, have been verified as LXRα/β and LXRα antagonists respectively and have also been shown to have hyperlipidemia lowering properties [48], [49]. Kanaya et al have reported that white button mushrooms have protective effects against liver steatosis through the inhibition of LXR signaling [50]. We show that EK selectively inhibits LXRβ transactivity in the presence of the LXRβ agonist GW3965, suggesting that EK contains an antagonist of LXRβ which competitively binds to LXRβ. The metabolic effect of LXRβ inhibition by EK is also shown on gene expression. The mRNA expression of LXRβ targets genes that control fatty acid oxidation, regulates fatty acid and cholesterol synthesis, such as ABCA1, ABCG1, LPL, and ApoE, and was significantly inhibited in the liver and fat tissue of EK treated mice. Identification of this specific LXRβ ligand may result in a novel therapy for metabolic diseases.

In conclusion, we provide evidence that EK protects against the development of obesity, hyperlipidemia and insulin resistance in high-fat diet-fed mice. These findings suggest that EK may be used as a potential dietary strategy for preventing metabolic disorders such as obesity, hyperlipidemia, diabetes and atherosclerosis. The potential of using naturally-occurring dietary supplements to regulate body weight and lipid metabolism is attractive. Because this traditional beverage is safe and cheap, it should be considered as a dietary therapy for metabolic syndromes. This is particularly important because weight loss and the treatment for non-alcoholic fatty liver disease have a poor long-term success rate. Further investigations are needed to define the mechanisms by which this component protects against obesity and its associated symptoms.

## Supporting Information

Figure S1 **Fingerprint Analysis of kuding tea extract by RP-HPLC.** A. Chromatograms of EK. Eluents were detected at 270 nm. **A**–C: Chromatograms of EK (B) and ursolic acid (C) with the gradient elution of HPLC was 48% A (acetonitrile) and 52% B (phosphoric acid, pH 2.5) at 0 min, 75% A and 25% B at 40 min, 85% A and 15% B at 60 min. D-E: Chromatograms of EK (D) and lupeol (E) with the constant mobile phase of methanol: water (98∶2, v/v). Eluents were detected at 210 nm for B-E.
(TIF)
