# Supplementary figures and images for: Extract of Kuding Tea Prevents High-Fat Diet-Induced Metabolic Disorders in C57BL/6 Mice via Liver X Receptor (LXR) β Antagonism
Source: PLoS One. 2012 Dec 4;7(12):e51007. doi: 10.1371/journal.pone.0051007 (PMC3514219; doi:10.1371/journal.pone.0051007)

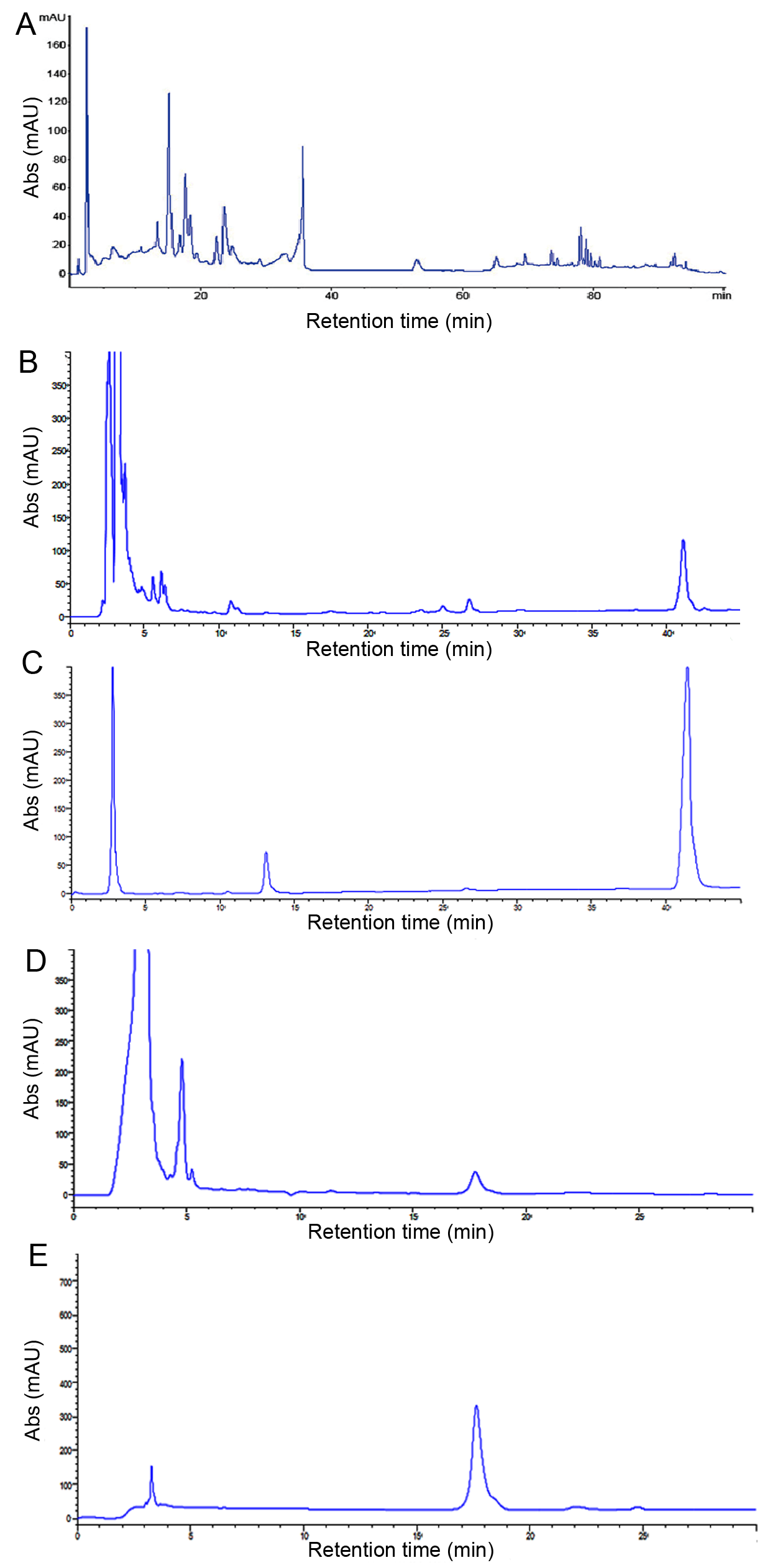

Supplement: Figure S1 — Fingerprint Analysis of kuding tea extract by RP-HPLC. A. Chromatograms of EK. Eluents were detected at 270 nm. A–C: Chromatograms of EK (B) and ursolic acid (C) with the gradient elution of HPLC was 48% A (acetonitrile) and 52% B (phosphoric acid, pH 2.5) at 0 min, 75% A and 25% B at 40 min, 85% A and 15% B at 60 min. D-E: Chromatograms of EK (D) and lupeol (E) with the constant mobile phase of methanol: water (98∶2, v/v). Eluents were detected at 210 nm for B-E. (TIF) [file pone.0051007.s001.tif]
